# From Load-Bearing Tissue to Failed Filter: A Conceptual Framework for Trabecular Meshwork Breakdown in Pseudoexfoliation Glaucoma

**DOI:** 10.3390/cells15151329

**Published:** 2026-07-24

**Authors:** Aparna Rao

**Affiliations:** Faculty Glaucoma Services, ICARE Eye Hospital and PG Institute, Noida 201301, UP, India; vinodini10375@yahoo.com

**Keywords:** trabecular meshwork, pseudoexfoliation glaucoma, pseudoexfoliation syndrome, intraocular pressure, aqueous outflow, extracellular matrix, TGF-β, LOXL1, outflow facility, glaucoma progression

## Abstract

Pseudoexfoliation glaucoma (PXG) is a unique cause of open-angle glaucoma with a more severe clinical course and higher intraocular pressure (IOP) than primary open-angle glaucoma (POAG). This reflects progressive structural failure of the trabecular meshwork (TM) driven by pseudoexfoliation (PXF) fibrillar material deposition, distinguishing PXG from POAG pathogenically. The TM, an active load-bearing filtration sieve, regulates IOP and is most affected by XFM deposition and extracellular matrix (ECM) dysregulation in PXG. Despite extensive research, the mechanism of trabecular dysfunction remains unclear, with both mechanical obstruction and ischemic/oxidative stress implicated. This narrative review amalgamates evidence across TM biology, PXF material science, and glaucoma pathophysiology to propose a five-stage framework for TM failure in PXG.

## 1. Introduction

Pseudoexfoliation glaucoma (PXG) represents a distinct form of open-angle glaucoma, with unique features of exfoliative material deposition over ocular structures, with a global prevalence ranging from 0 to 80% [1,2,3]. An estimated 60–70 million individuals are projected to have pseudoexfoliation syndrome (PXF), of whom approximately one-third develop glaucoma [1,2]. It is well known that PXG has a more aggressive clinical course than primary open-angle glaucoma (POAG), presents with a higher intraocular pressure (IOP), and has greater diurnal IOP fluctuations, faster rates of visual field (VF) progression, poorer response to medical therapy, and higher intraoperative complications [1,3,4,5,6,7,8]. These unique features are attributed to the pathogenesis of XFM that involves aberration of ECM homeostasis and deposition of XFM material, both of which can have multiple triggers like ischemia and oxidative stress [4].

The TM is an active, mechanosensory, contractile tissue with an intricate balanced relationship with the surrounding extracellular matrix (ECM). Dynamic homeostasis between these two vital components under physiological conditions is maintained though several pathways and key molecules that regulate ECM degradation, autophagic clearance of debris and collagen cross-linking [4,9,10]. When these regulatory mechanisms are compromised or overwhelmed, TM contractility is reduced, causing accumulation of debris, which causes progressive TM cell death and tissue failure [8,9].

In PXG, the accumulation of PXF fibrillar material, a unique protein-complex aggregate of elastic microfibrils and matrix proteins with carbohydrate moieties, within the juxtacanalicular tissue (JCT) and the corneoscleral meshwork causes progressive mechanical obstruction and increase in TM stiffness [8,9,10,11]. Therefore, in PXG, the TM faces an added onslaught of a mechanical burden caused by XFM deposits, with oxidative stress, ischemic/vascular triggers, and pro-fibrotic milieu, causing a severe clinical phenotype as compared to POAG [4,8,10,11,12].

This fundamental change in the TM in PXG from an active filter to a failed, obstructed tissue prompts the question of what is more important in PXG management: “lowering IOP alone?” rather “preventing TM structural failure?” Previous studies have predominantly focused on PXF epidemiology, clinical features, glaucoma risk, or postoperative complications. Despite years of research, the actual mechanism of TM damage in PXF remains unexplored, owing to the difficulty in studying temporal changes in TM tissue in patients, or the lack of an animal model for PXF [8,9,10,11,12]. Very few studies focus on ultrastructural changes in TM specimens directly obtained from patients with the disease that depict differences in TM damage in PXG compared to other forms of glaucoma [11]. The present review amalgamates evidence from TM biology, PXF molecular pathology, and impaired aqueous outflow physiology to construct a framework for reasons of TM failure in PXG. It also discusses how this framework compares with POAG pathophysiology, and outlines the therapeutic implications of staging TM dysfunction as a primary outcomes measure (rather than IOP) of glaucoma management.

Methods: We screened PubMed to identify articles discussing TM damage in PXF/PXG or POAG using the search strategy (((Trabecular meshwork) AND (Exfoliation glaucoma)) AND (primary open angle glaucoma)) OR (pseudoexfoliation), restricting articles published from 2000 to 2026. All abstract articles in English were screened for mechanisms of TM damage with no inclusion or exclusion criteria used.

## 2. The Normal Trabecular Meshwork: A Load-Bearing Filtration Sieve

### 2.1. Lamellar Architecture and Layer-Specific Function

The TM is an ultrastructural sieve located at the inner angle of the anterior chamber between the iris, ciliary body and the lens, spanning anatomically from the termination of Descemet’s membrane anteriorly to the scleral spur posteriorly [10,12,13,14,15,16,17,18,19,20,21,22,23,24,25]. The three TM layers, namely the uveal, corneoscleral, and juxtacanalicular (JCT), contribute to distinct functional properties that regulate aqueous outflow and are vital for IOP maintenance under physiological conditions.

The uveal and corneoscleral layers consist of interlaced collagen beams lined by TM endothelial cells, forming a series of TM sheets with inter-trabecular spaces that differ in dimensions within the three layers. It is through these spaces that aqueous humour percolates into collector channels and Schlemm’s canal [10,14,16]. The critical site of outflow resistance resides in the JCT with the tightly packed TM cells with the narrowest IT spaces, and lies immediately beneath the inner wall endothelium of Schlemm’s canal (SC) [14,15,16,17,18,19,20,21,22,23]. The JCT comprises a loose network of fibrillar ECM through which TM cells extend filopodial processes and maintain contact with each other and with the SC [10,14,16,17,22]. Current evidence suggests that the JCT is the key region affected in PXG; however, ultramicroscopic features suggest the involvement of the CSM and uveal meshwork, though the precise molecular changes within each region in PXG, is unexplored [8,11,16].

### 2.2. TM-ECM Matrisome

TM cells express alpha-smooth muscle actin (α-SMA), myosin light chain kinase, and Rho-associated kinase (ROCK), along with expression of molecules involved in cytoskeletal contractile machinery, which are key for modulation of the cell dimensions and inter-trabecular spaces in response to mechanical and other external stimuli [10,11,24,25,26,27,28]. This cytoskeletal contractility increases outflow facility which comprises the functional role of TM cell tone in resistance generation [10,11,14,21]. The TM cells under physiological conditions phagocytose debris, pigment granules, erythrocyte or cell debris, and fragments of ECM that accumulate in the aqueous humour, maintaining patency of the outflow pathway [10,14,16]. This is aided by the UPR pathway molecules that regulate autophagic clearance of debris and EM breakdown products to ensure TM contractility is maintained for adequate egress of aqueous. This phagocytic activity is ATP-dependent and declines with age, parallel to the reduction in TM cell density [8,14,16,28]. In PXF syndrome and pigmentary glaucoma, chronic phagocytic overload exhausts this capacity, initiating the cascade of structural failure [8,28].

### 2.3. ECM Homeostasis and Outflow Regulation

The JCT ECM remodelling is mediated by matrix metalloproteinases (MMPs), their tissue inhibitors (TIMPs), lysyl oxidases, and a range of growth factors including TGF-β, fibroblast growth factor (FGF), and connective tissue growth factor (CTGF) [10,14,17,29,30,31,32,33]. TM cells constitutively express MMP-1, MMP-2, MMP-3, and MMP-9, whose activity is regulated by growth factors, mechanical strain, and pharmacological agents [4,6,7,8,29,30,31,32,33,34,35]. Under elevated IOP, MMP expression is upregulated, which reduces ECM density and increases outflow facility [28,29,30]. TGF-β2, the predominant aqueous isoform, is a master regulator of JCT ECM composition with a dual role in glaucoma [31,32,33]. In the earlier stages of PXF and in normals, TGF upregulation helps maintains homeostasis and TM cell repair, while in chronic glaucoma, divergence in the TGF downstream pathways causes a pro-fibrotic milieu causing TM stiffness/cell death [28,31,32,35,36,37]. This is often referred to as the “TGF paradox” which seems to explain differential expression of key ECM molecules, like LOXL1, in PXF and PXG. In normal ageing, there is a gradual decline in outflow facility associated with increased JCT ECM density, reduced TM cell density, and decreased MMP activity, which causes progressive increase in TM stiffness and loss of TM function [29,30,31,32,33,34,35,36,37].

### 2.4. Mechanosensory Function: The TM as an IOP Sensor

The TM cells sense and respond to changes in IOP-generated mechanical strain via integrin-mediated ECM signalling, stretch-activated ion channels, and cytoskeletal remodelling [9,10]. Mechanosensors are believed to exist in the scleral spur, yet TM cells sense mechanotransduction via ion-gated channels. Shear or stretch by IOP causes Piezo1 activation, triggering downstream processes that causes TM relaxation, improving outflow facility [38,39,40,41,42]. TRPV4 channels regulate Ca-driven signalling cascades that cause eNOS and NO production within the conventional outflow pathway, with feedback loops with Rho signalling to regulate cytoskeletal remodelling, required for TM contractility [41,42]. These molecules are key for maintaining the TM tone and the IOP and regulate how TM/SC behave in response to external stressors like TGF and ECM composition [23,25,39]. The TM cells actively share information with the ECM though transmembrane proteins, integrins, that connect with the actin-myoskeleton within TM cells and sometimes to the intermediate filaments [10,38]. ECM turnover is regulated by the balanced activity of matrix metalloproteinases (MMPs) and tissue inhibitors of metalloproteinases (TIMPs) [30,32,33]. In response to raised IOP, TM cells upregulate MMP activity, which helps in EMC degradation and improving outflow [32,33]. Regulation of cross-linking of ECM elastin and collagen through LOXL1 further helps regulate ECM composition and density [43,44,45,46,47,48,49]. The constant feedback between the TM/SC complex and the ECM (via integrins and other transmembrane proteins) works as a syncytium (matrisome), which is key for maintaining the outflow facility and IOP under physiological conditions and under different stressors [4,16,28,29]. Disruption of these vital sources of information of mechanosensing owing to cell loss, cytoskeletal stiffening, or ECM rigidity converts the TM from an active pressure regulator to a fixed-resistance vestigial tissue. In PXF, MMP9 activity is found to be increased, which, however, reverses with poor activity seen in PXG. This does not merely represent a lack of information on mechanosensing, but an overwhelming amount of compensatory mechanisms owing to ECM rigidity and XFM deposition in PXG [30,32]. This partly explains the higher IOP at the same severity, and the differences in clinical features that make PXG unique among glaucoma forms with systemic features [2,3,5,32,40,50,51,52,53,54,55,56,57,58,59]. This specifically means that PXG represents a disease wherein TM dysfunction, at least in the early stages, is not from an intrinsic failure of mechanosensing but exhaustion of clearance pathways [28,32,33]. This suggests that interventions targeted at reducing XFM and enhancing clearance pathways may help control the IOP more effectively than conventional TM-based surgeries; additionally, addressing the ECM rigidity may help restore TM function in PXG in contrast to other glaucoma forms.

## 3. Pseudoexfoliation Material: Origin, Composition, and Trabecular Tropism

PXF material is a unique pathological aggregate that cannot be reproduced experimentally, which reflects its complex, multi-component biosynthesis, amidst an ageing TM/SC-ECM complex influenced by specific triggers like TGF, oxidative stress and ischemia [56]. LOXL1 variants disrupt the normal cross-linking and assembly of elastic microfibrils, leading to the accumulation of incompletely cross-linked elastic-like protein-complex aggregates unique to PXF [29,43,44,45,46]. Recent genome-wide association studies have reported additional loci like CACNA1A, TMEM136, AGPAT1, and SEMA6A, which suggests a multifactorial aetiology in PXG involving calcium signalling, lipid metabolism, and ECM remodelling, compared to merely elastic fibre pathology [60]. At the ultrastructural level, PXF fibrils comprise a core of fibrillin-1 and fibrillin-2 microfibrils, surrounded by glycoconjugates including heparan sulphate, dermatan sulphate, and chondroitin sulphate and carbohydrate moieties [28,29,56]. Also contained within this matrix are other components including clusterin, vitronectin, apolipoprotein E, complement factors, and amyloid P components [56]. The aberrant cross-linking by LOXL1 confers PXF fibrils resistance to proteolytic degradation, posing a difficulty for clearance mechanisms like autophagy and UPR pathways that are unable to clear these complexes from the ECM [28,29,31]. While XFM accumulates in all regions of the TM, JCT, with the highest resistance and smallest IT spaces, suffers maximum dysfunction [8,11]. The ECM in the JCT region contains fibronectin and heparan sulphate proteoglycans which have a higher binding affinity for the glycoconjugate complexes found in PXF fibrils. Ultrastructural studies revealed that changes are seen in all three TM regions [11]. Logically, the JXT is expected to be more affected in PXG. Yet the fact that all three regions are fully affected suggests that the pathophysiology in PXF/PXG is diffuse with individual susceptibility only depending on IT spaces that determine maximum obstruction to outflow rather than functional damage necessarily in all regions, at least in the earlier stages. Chronic XFM deposition now affects TM contractility and changes in ECM compositions creating a pro-fibrotic milieu that causes deranged TM contractility and then eventually cell death [4,11,31,43,54,56,61,62,63,64,65].

We present below a conceptual framework of TM dysfunction in PXG that occurs in stages. Table 1. Transition from one stage to the other at the molecular levels happens slowly and is currently clinically unrecognisable unless seen as raised IOP or progressive disc damage. Understanding all levels of damage of the TM in PXG would help us understand the reasons for progression, which would enable adoption of appropriate tailored corrective measures.

## 4. Stages of TM Dysfunction in PXG

### 4.1. Stage I: SOS Signal Due to Debris Overload

In the earliest phase of TM involvement in PXG, PXF fibril accumulation within the inter-trabecular spaces of the corneoscleral meshwork and, specifically, within the JCT causes impedance to aqueous egress and thus initiates a cascade of events meant to clear this debris. Quantitative morphometric studies have demonstrated a significant reduction in open pore area in PXG TM specimens compared with age-matched controls, with the most pronounced changes at the inner wall of Schlemm’s canal [8,10,11,54,64,65]. At this stage, TM cellularity may be relatively preserved with preserved TM contractility and increased phagocytic activity. At the molecular levels, this means activation of autophagic clearance of XFM aggregates, ECM remodelling, upregulation of the UPR pathway, and increase in MMP activity causing increased EMC degradation [31,32]. Also reported as the earliest event is breakdown of the blood–aqueous barrier, though this is difficult to monitor clinically [51].

The clinical signature of Stage I is either a phase of normalcy or an IOP asymmetry (either presenting or on diurnal phasing) that exceeds what would be expected from aqueous production differences alone. This may also be accompanied by reduced ONH perfusion or an asymmetric decrease in RNFL thickness in one eye [66,67,68].

### 4.2. Stage II: Cellular Exhaustion

**Figure 1:**
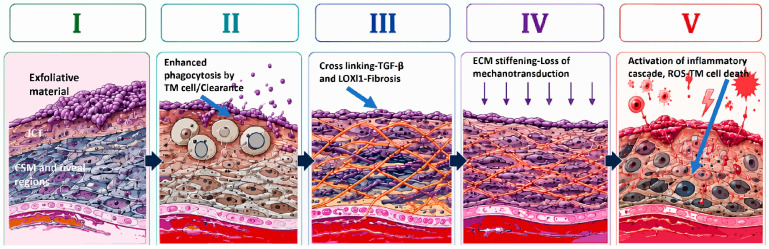
Conceptual stages of trabecular meshwork failure in pseudoexfoliation glaucoma; see text for detailed description of each stage.

As PXF fibril accumulation continues, TM cells mount a phagocytic response that initially serves to clear material from the inter-trabecular spaces. However, the phagocytic capacity in PXG is overwhelmed by chronic disease and progressive deposition of aggregates, which are rendered insoluble by anatomical cross-linking and polymerisation [29,31]. Alvarado and colleagues demonstrated TM cell loss in POAG that is accelerated in PXG, with cell densities falling below the minimum required for ECM homeostasis at an earlier age [65]. Progressive accumulation of these deposits now exerts a direct toxic effect that causes TM cell death that only hastens age-related cell apoptosis in the tissue, further ushering the disease into irreversibility. TM cells are the sole producers of the MMPs and growth factor modulators required for ECM remodelling. A pro-fibrotic milieu is created, with fibronectin and tenascin accumulation, and excessive unregulated collagen cross-linking, with reduced capacity for adaptive ECM turnover, activation of inflammatory cascades, and accumulation of ROS [28,29,32]. So this causes the vicious cycle to be started with more PXF material accelerating cell death and fewer cells now permitting greater fibril accumulation. The transition from Stage I to Stage II marks the beginning of this self-reinforcing cycle, depicted in Figure 1.

### 4.3. Stage III: Fibrotic Dysregulation

Under normal conditions, TGF-β1 and TGF-β2 are present in aqueous humour and exert tonic influences on TM ECM composition, balanced by anti-fibrotic factors including decorin, biglycan, and hepatocyte growth factor (HGF) [47,48]. Aqueous TGF-β1 levels are significantly elevated compared to both age-matched controls and POAG eyes, and TGF-β2, the dominant isoform in POAG, is also upregulated [49,50]. In PXG, this balance is disrupted with dysregulation of TGF downstream processes. Downstream of TGF-β signalling, CTGF (CCN2) is upregulated in PXG TM, driving the synthesis of collagen I and collagen III and promoting their deposition in the JCT [51,52]. Others like fibulin, fibronectin, and LOXL1 are dysregulated owing to differential effects of continued TGF upregulation, something referred to as TGF paradox in PXG. This causes the ECM to become progressively stiffer and more resistant to MMP-mediated degradation [53,54]. Though MMP9 protein is found to be increased reflecting increased ECM degradation, MMP9 activity is reported to decline to severe stages of PXG despite continued TGF upregulation, again reflecting divergence and dysregulation of downstream pathways. Stage III therefore may represent a transition toward structural irreversibility that is qualitatively distinct from the functional outflow resistance of earlier stages.

### 4.4. Stage IV: Mechanosensory Failure

The mechanosensory function of the TM depends on the back-and-forth feedback transmission of mechanical signals between cells and ECM through integrins [55,56]. With a stiff fibrotic ECM seen in Stage 2, TM cells transform into a myofibroblast-like phenotype (epithelial–mesenchymal transformation), and paradoxically increase contractility [57,58]. If the trigger continues as seen in untreated/uncontrolled disease, this mechanosensory feedback loop is attenuated or broken, as depicted in Figure 1. Therefore, IOP changes during physiologic conditions like cardiac pulsation, posture, and diurnal aqueous production fail to be compensated by outflow facility adjustment. This also activates other cascades like oxidative stress or mitochondrial damage [4,32,59,60]. This may be the reason why unilateral PXF is considered to be bilateral PXF in transition, with the clinically normal eye undergoing changes at earlier stages that have not manifested clinically [66,67]. The therapeutic implication at Stage IV may be that the mechanism of Rho kinase inhibitors or other drugs targeting the TM/SC complex may be ineffective because of mechanosensory uncoupling, thereby requiring alternate measures to stop disease progression. This also means that studies evaluating the TM/SC complex under physiological conditions like diurnal phasing or appropriate provocative tests may give insights into early transition of normal contractility in the earlier stages to transition to Stage 3.

### 4.5. Stage V: Inflammatory Amplification

**Figure 2:**
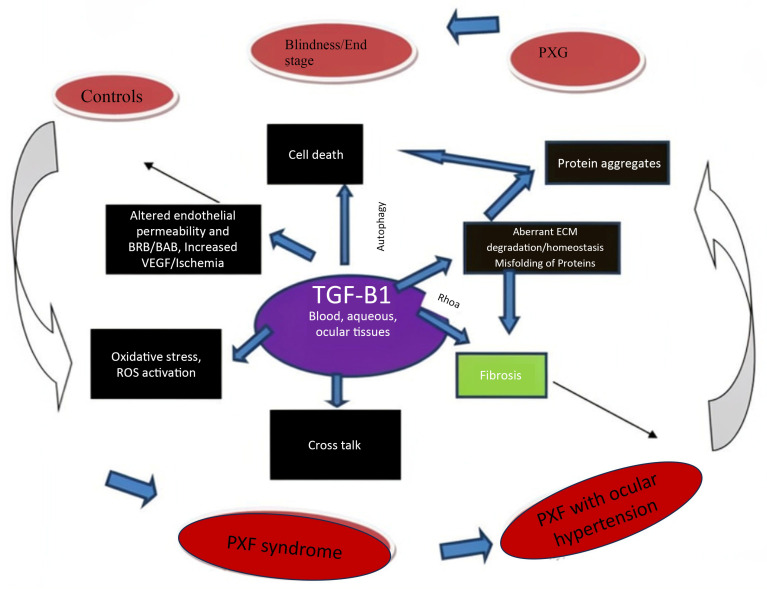
The TGF paradox in pseudoexfoliation and pseudoexfoliation glaucoma; see text for full description.

The last stage of the cascade in TM tissue involves a transition from a stage of structural and biomechanical alterations to a vicious self-perpetrating inflammatory microenvironment. Several studies support a significant oxidative and inflammatory component in PXG, with breakdown of the blood–aqueous barrier seen early in the disease process [4,6,7,8,15]. PXG eyes have been reported to have markedly elevated levels of aqueous 8-hydroxy-2′-deoxyguanosine (8-OHdG), malondialdehyde, and protein carbonyls, alongside depleted antioxidant defences including glutathione and catalase [31,34,51,69,70]. Mitochondrial dysfunction in TM cells reduces ATP production and impairs phagocytosis and autophagic clearance mechanisms, causing apoptosis of cells and loss of function [4,29,31]. Simultaneously, NF-κB activation in PXG TM stimulates pro-inflammatory cytokines like IL-6, IL-8, and TNF-α, which in turn upregulate TGF-β signalling, promoting TM cell apoptosis, which further amplifies the inflammatory environment, as shown in Figure 2 [71].

This inflammatory loop is largely self-sustaining once established. Oxidative stress activates NF-κB which promotes TGF-β and CTGF-induced fibrosis, causing ECM stiffness, TM cell myofibroblast transformation, and more oxidative stress, closing the self-perpetuating loop. In this stage, uncontrolled IOP may be more common with clinical refractoriness to medical therapy and fast progression, despite treatment. Here, surgical procedures may actually bypass the TM relieving the IOP since the TM mechanical and structural failure renders it ineffective in its functions in Stages 1 and 2. Since now these processes continue irrespective of fibril deposition that triggers the events eventually, these stages may represent why IOP in PXG is higher than POAG of similar disease severity. Since the molecular events may have progressed to later stages of TM irreversible dysfunction, the extent of PXF material does not correlate clinically with IOP or gonioscopic findings. Post mortem histological studies of PXG TM have consistently demonstrated reduced cellularity, increased JCT ECM density, collapsed Schlemm’s canal, and fibril deposition across all layers of the meshwork, with changes most pronounced in the JCT [3,4,8,11,61]. The reduction in aqueous outflow in PXG correlates poorly with IOP level measured at a single time point, suggesting that resistance generation is not simply a function of IOP-driven fluid dynamics or mechanical obstruction by XFM, but reflects the consequence of fibrotic and mechanosensory failure in later stages. In individuals with unilateral PXF, the affected eye demonstrates lower outflow facility, higher IOP, and a greater diurnal fluctuation, despite an identical genetic background [66,67,68]. The unilateral eye provides a “precursor” of earlier stages at the tissue level as compared with the advanced bilateral PXG eyes [67]. This again suggests that genetic markers for PXFG/PXG may not reflect the true tissue-level staging that is important for appropriate prognostications and guiding clinical decisions. This also explains partly why markers of epigenetic regulation in the anterior chamber (tissue expression in aqueous, iris, TM or ciliary body) in each eye are a more specific indicator of true dysfunction or stage of disease pathogenesis than blood-based biomarkers [72,73].

## 5. Comparison with Primary Open-Angle Glaucoma: Shared Endpoints and Distinct Mechanisms

PXG and POAG converge on the same structural endpoint of TM cell loss and tissue fibrosis with elevated outflow resistance, yet these milestones are reached via distinct pathways/genes and at different rates between PXG and POAG [16,54,58,61,74]. This distinction is essential for avoiding the clinical error of treating PXG as simply a more severe form of POAG. Table 2 summarises the principal differences.

### TGF-β Signalling: Convergent Fibrosis and Divergent Triggers

TGF-β2, the dominant pro-fibrotic cytokine in POAG, is elevated in aqueous humour, and regulates ECM accumulation in the JCT through SMAD2/3-mediated transcription of collagen and fibronectin [33,34,35,36]. In PXG, both TGF-β1 and TGF-β2 are elevated, with TGF-β1 associated with inflammatory and wound-healing fibrosis disproportionately, being elevated [33]. This suggests that the triggers and processes responsible for fibrosis in PXG are different. In POAG, fibrosis is possibly an intrinsic process of ECM dysregulation, while in PXG, fibrosis is predominantly driven by the presence of PXF material acting as a chronic inflammatory and mechanical stimulus, as in Figure 2. The LOXL1-mediated cross-linking compounds the mechanical consequences of fibrosis far beyond what is seen in POAG, making PXG unique [45,46,48]. Studies have reported cell densities of approximately 300–400 cells/mm^2^ in end-stage POAG, compared with 750–1000 cells/mm^2^ in age-matched controls [29,54,65]. In PXG, cell density reductions occur at an earlier age, owing to the additional phagocytic burden and oxidative stress of Stages II and V [29,54]. This may explain partly the severe clinical parameters at similar clinical severity in PXG compared to POAG.

## 6. Early Surgical Intervention: Structural Bypass vs. TM Rescue

Current surgical management of PXG like trabeculectomy, tube surgery, and minimally invasive glaucoma surgery (MIGS) achieves IOP control by creating alternative aqueous outflow pathways and bypassing the TM/SC complex rather than by rescuing the failed TM. This is clinically effective in terms of IOP control, but leaves the underlying TM pathology unaddressed. Two implications follow from the staged failure framework. First, surgical intervention should be considered earlier in PXG than in POAG, for example at Stages II–III rather than after medical failure is well established. Second, MIGS procedures that engage the TM directly may be rendered less effective as TM failure advances, though this needs longitudinal studies on PXG cohorts with different severity of damage.

Several critical questions remain unanswered in PXG. The most pressing question is that of what defines the “point of no return” beyond which corrective therapies may be insufficient to restore meaningful outflow capacity, and which signifies transition to PXG from PXF. Though this framework provides a possible mechanism of gradual TM damage in PXF, this is just a conceptual framework that needs validation with in vivo studies and studies on animal models studying TM changes with time. Furthermore, multiple triggers like inflammation or oxidative stress may act on the TM simultaneously. This framework may guide us to the possible mechanisms of TM damage in a stepwise manner, although it cannot replicate real in vivo changes where multiple mechanisms may cause TM dysregulation simultaneously. Therefore, identifying the point of no return in vivo currently is a major challenge that requires animal models of PXG, which are currently non-existent. Furthermore, since blood-based markers do not indicate tissue-level changes perfectly in any ocular condition, can aqueous humour or tear biomarkers, including PXF fibril burden, LOXL1 protein levels, TGF-β1/β2 concentrations, epigenetic markers, or oxidative stress indices, provide a non-invasive tool that can mark the change to PXG at the tissue level? Lastly, is there a therapeutic window for fibril dissolution in vivo, or are there safe methods of LOXL1 inhibition that could arrest the transition from Stage II to Stage III? Nevertheless, PXG represents the effect of a continuum of changes causing severe TM dysfunction caused by multiple triggers. This framework may help clinicians to identify possible clinical indicators of disease progression in early disease, while helping in surgical decision-making. More TM-based research focusing on local biomarkers is essential to capture these stage transitions, which would guide clinicians in understanding the true risk of disease progression and making appropriate clinical decisions.

## Figures and Tables

**Table 1:** Proposed five-stage framework for trabecular meshwork failure in pseudoexfoliation glaucoma.

Stage	Designation	Dominant Pathologic Process	Clinical Correlates	Histological/Correlates	Molecular Biomarkers	Imaging Correlates
I	Physical overload	PXF fibril accumulation in JCT, pore occlusion, raised outflow resistance without cell loss	Reduced C-value, early IOP asymmetry [1,3,4], TM pigmentation on gonioscopy	PXF fibrils in ECM/TM/TM [11,28]	TGF β1 upregulation, LOXL1 [8], upregulation, enhanced MMP (enzyme activity) [32,33,34,35]	None
II	Cellular exhaustion	Phagocytic overload, vacuolar accumulation, lipofuscin deposition, TM cell apoptosis begins	Pupillary ruff atrophy, raised IOP or diurnal fluctuations [66,67]	PXF fibrils in ECM/TM with morphological TM changes in CSM, declining cell density on histology [1,8,11]	Enhanced UPR pathway [29,30,31,32]	TM thinning on AS-OCT [1,30]
III	Fibrotic dysregulation	Unopposed TGF-β1/β2 signalling, collagen I/III deposition, LOXL1-mediated ECM cross-linking	Poor medication response [1,2,3]	ECM fibrosis, extensive fibril deposition, EMT changes, reduced density and EMT changes in CSM > JXT [11]	Increased ECM proteins, LOXl1 downregulation, reduced MMP9 activity despite TGF β1 upregulation [28,29,30,31,32]	Hypertrophic scar [1,3]
IV	Mechanosensory failure	Stiffened TM/ECM decouples TM cells from IOP signals, loss of cytoskeletal pressure-buffering	Disc/Vf changes, large peak–trough fluctuation [66,67,68]	Severe EMT changes in TM, ECM fibrosis, reduced TM contractility [11,28]	Undetectable MMP9 enzyme activity, reduced UPR response, elevated cytokines like IL-6 and IL-8 [8,28]	Hypertrophic scar on ASOCT [1,3], disc/peripapillary changes [2,3,67,68]
V	Inflammatory amplification	NF-κB activation, IL-6/TNF-α upregulation, oxidative stress, mitochondrial dysfunction, self-sustaining cell loss	Accelerated VF loss, high surgical conversion rate [1,2,3,4,58]	Diffuse changes across all regions of the TM, extensive EMT changes and TM thinning [3,8,11]	Markedly elevated 8-OHdG, elevated cytokines like IL-6, IL-8 and TNF-α [3,8]	

JCT = juxtacanalicular tissue; ECM = extracellular matrix; TGF-β = transforming growth factor beta; LOXL1 = lysyl oxidase-like 1; AS-OCT = anterior segment optical coherence tomography; IOP = intraocular pressure; VF = visual field; NF-κB = nuclear factor kappa-light-chain-enhancer of activated B cells; EMT = epithelial–mesenchymal transformation; UPR = unfolded protein response; IL = interleukin; TNF-α = tumour necrosis factor alpha. Note: The parallel histopathological, imaging, molecular signatures, and clinical correlates are speculative only in each stage.

**Table 2:** Comparison of TM failure characteristics in PXG and POAG.

Parameter	PXG	POAG
Primary TM insult	Exogenous PXF material + intrinsic ECM dysregulation	Intrinsic cytoskeletal stiffening; ECM accumulation
IOP level	Higher; greater diurnal fluctuation	Variable; often moderate
IOP asymmetry	Pronounced; correlates with unilateral PXF load	Mild to absent
Fibrotic pathway	TGF-β1/β2 + LOXL1-amplified cross-linking	TGF-β2-dominant; slower progression
Oxidative burden	Higher; systemic component	Moderate; largely localised
Rate of VF progression	Faster; steeper MD decline	Slower; more stable
Medical therapy response	Poorer; structural resistance predominates	Better; functional resistance predominates
Surgical conversion rate	Higher; earlier	Lower; later

PXG = pseudoexfoliation glaucoma; POAG = primary open-angle glaucoma; TM = trabecular meshwork; ECM = extracellular matrix; IOP = intraocular pressure; VF = visual field; MD = mean deviation; TGF-β = transforming growth factor beta; LOXL1 = lysyl oxidase-like 1.

## Data Availability

No new data were created or analyzed in this study. Data sharing is not applicable to this article.
